# SENESCENCE-ASSOCIATED SECRETOME COMPONENTS AS BIOMARKERS FOR AGING

**DOI:** 10.1093/geroni/igz038.369

**Published:** 2019-11-08

**Authors:** Xu Zhang, Marissa Schafer, Thomas White, Sarah Jachim, Nathan LeBrasseur

**Affiliations:** 1 Mayo Clinic, Rochester, Minnesota, United States; 2 Robert and Arlene Kogod Center on Aging, Mayo Clinic, Rochester, Minnesota, United States

## Abstract

Cellular senescence is a state of stable growth arrest in response to stress, which is a fundamental process of biological aging. They secrete products, the Senescence-Associated Secretory Phenotype (SASP), which consists of inflammatory cytokines, chemokines, growth factors and matrix remodeling proteins. Senescent cells accumulate with advancing age and partial elimination of senescent cells can reverse age-related dysfunction and increase mean lifespan in mice. However, it is not clear whether components of the SASP can be measured in human plasma and serve as aging biomarkers. Here we generated a candidate panel of senescence markers based on a multiplexed bead-based assay of proteins secreted by senescent preadipocytes, endothelial cells, fibroblasts, myoblasts, preadipocytes, and epithelial cells compared to non-senescent controls. The SASP undoubtedly varies by cell type; however, we observed that multiple components of the SASP are conserved. We then assessed circulating SASP components in human plasma samples from Mayo Clinic Biobank participants (n=280, 20 male and 20 female per decade, age 20-90) using the same method. We confirmed that components of the SASP can be quantified in human plasma with the multiplexed bead-based assay and observed several SASP components robustly increase with chronological age in humans. Our study illustrates that senescence-associated secretome components are detectable in human plasma and could potentially serve as biomarkers of systemic aging and senescent cell burden.

